# Automated Size Recognition in Pediatric Emergencies Using Machine Learning and Augmented Reality: Within-Group Comparative Study

**DOI:** 10.2196/28345

**Published:** 2021-09-20

**Authors:** Michael Schmucker, Martin Haag

**Affiliations:** 1 GECKO Institute Heilbronn University of Applied Sciences Heilbronn Germany

**Keywords:** resuscitation, emergency medicine, mobile applications, mobile phone, user-computer interface, augmented reality, machine learning

## Abstract

**Background:**

Pediatric emergencies involving children are rare events, and the experience of emergency physicians and the results of such emergencies are accordingly poor. Anatomical peculiarities and individual adjustments make treatment during pediatric emergency susceptible to error. Critical mistakes especially occur in the calculation of weight-based drug doses. Accordingly, the need for a ubiquitous assistance service that can, for example, automate dose calculation is high. However, few approaches exist due to the complexity of the problem.

**Objective:**

Technically, an assistance service is possible, among other approaches, with an app that uses a depth camera that is integrated in smartphones or head-mounted displays to provide a 3D understanding of the environment. The goal of this study was to automate this technology as much as possible to develop and statistically evaluate an assistance service that does not have significantly worse measurement performance than an emergency ruler (the state of the art).

**Methods:**

An assistance service was developed that uses machine learning to recognize patients and then automatically determines their size. Based on the size, the weight is automatically derived, and the dosages are calculated and presented to the physician. To evaluate the app, a small within-group design study was conducted with 17 children, who were each measured with the app installed on a smartphone with a built-in depth camera and a state-of-the-art emergency ruler.

**Results:**

According to the statistical results (one-sample *t* test; *P*=.42; α=.05), there is no significant difference between the measurement performance of the app and an emergency ruler under the test conditions (indoor, daylight). The newly developed measurement method is thus not technically inferior to the established one in terms of accuracy.

**Conclusions:**

An assistance service with an integrated augmented reality emergency ruler is technically possible, although some groundwork is still needed. The results of this study clear the way for further research, for example, usability testing.

## Introduction

### Background

The results in pediatric emergencies are not satisfactory. Too few children survive such emergencies with favorable neurological outcomes [1,2]. There are several reasons for this. First, this type of emergency is very rare; in Germany, for example, there are only 1000 prehospital resuscitations for 30,000 emergency physicians per year, that is, on average, one emergency physician resuscitates a child every 30 years [3]. This observation deliberately ignores that there are specially trained pediatric emergency physicians in urban areas, as pediatric emergency physicians are not common. Second, emergency physicians find it difficult to remain calm in a child emergency. In a survey of 104 emergency physicians, conducted by Zink et al [4], 88% said that they had already felt anxiety or excessive pressure at work. When asked for the reason, 84% said they had experienced these feelings in a pediatric emergency, followed by polytraumatized patients (20%) and obstetric emergencies (18%). Multiple answers were possible. Apart from the fact that the patient is a child and the psychological consequences that may result, it is mainly the anatomical differences between children and adults and the associated peculiarities of resuscitation that cause problems for emergency physicians. Although the resuscitation of an adult is quite standardized, there are individual differences in every child. The choice of using different processes as well as equipment is influenced by the size or weight of the child. For example, the size of the endotracheal tube and depth of insertion are specified [5] or the dosage of medication is calculated individually based on the patient’s weight [6]. Especially in drug dosing, mistakes happen rather frequently, sometimes with life-threatening consequences [7-9]. This is because it is difficult to determine a child's weight and thus the correct dose. There are various methods to determine a child’s weight. As Young and Korotzer [10] describe in their systematic analysis, the most precise method is parental estimation. If the parents are not present, the state of the art is to derive the weight from the height of the child using a so-called emergency ruler (eg, Broselow tape [6]). These tools are important, because the medics’ estimations are not very accurate, according to the systematic analysis mentioned earlier [10]. Despite these aids, emergency physicians repeatedly express a desire for technical aids [7,8]. Therefore, one idea is to create a ubiquitous assistance service that uses modern wearables (eg, a smartwatch for measuring the compression depth [11], head-mounted displays [HMDs] as screens or for telemedical scenarios [12,13]) to provide a service that requires as little attention as possible while still providing great assistance (principle of calm technology [14]). To accomplish this, a high degree of automation must be achieved in addition to a high level of usability. The idea is to recognize the patient with computer vision algorithms and to be able to measure the patient directly using a depth camera. All other parameters can then be automatically derived, calculated, and displayed on an HMD, for example, as integrated into the process steps of the American Heart Association [15] or the European Resuscitation Council (ERC) [16] guidelines. After literature research, expert interviews, and initial research results [17], an app was programmed and evaluated using a comparative study to apply this level of automation.

### State of the Art

There are several approaches to replace emergency rulers using technical support, for example, with a smartphone or a tablet [18-24]. Promising studies have already confirmed that the use of an app can minimize errors [22-24]. However, the problem with most apps (all mentioned earlier but one) is that there is no automation of size recognition, that is, manual entries are necessary. Apart from usability, there is also the problem that these values (age, weight, or height) must be known first. For inhospital cases, it can be assumed that the weight of the child is known; however, this does not apply to prehospital cases. For some apps, even the now-obsolete age-based formula for calculating the dosages is used [18], which is inferior to the length-based method [10]. A very interesting app is Optisizer developed by Wetzel et al [21]. A 20×20-cm tag placed next to the child is used as a reference value for the size. A first clinical trial looks promising [21]. However, the tag must be at the same level as the child, and the measurement must always be taken at a 90° angle. This is simply because a camera without additional sensor technology has no relationship to angle and depth, and therefore, an accurate calculation cannot be made automatically. A revised version of this app, which should solve this problem, is announced by the authors in the outlook [21].

The aim of this paper is therefore to fill this gap. An app is programmed and evaluated that uses augmented reality (AR) and a depth camera to provide a simple, fast, and safe way to automatically determine a child’s weight and thus the medication dosage.

## Methods

### Background

The evaluated app is based on a prototype in which the measuring accuracy of the Asus ZenFone AR’s depth cameras has already been proven [17]. This does not deviate significantly from the measurements made with the aid of an emergency ruler. However, the handling was problematic; the individual measuring points (head and foot of the child) had to be marked manually. To address this problem, the app was further developed so that it recognizes the child using machine learning and then performs an automated measurement. Furthermore, dosages for adrenalin and amiodarone are calculated and made available to the user. The calculations are based on the data of the KiGGS (*Studie zur Gesundheit von Kindern und Jugendlichen in Deutschland* [German Health Interview and Examination Survey for Children and Adolescents]) study of the Robert Koch Institute (RKI) [25] and the formulas stated in the ERC guidelines [16]. It must therefore be evaluated whether the new functionality of the app can repeat the good values of the previous study. The decisive factor here is how well the process of machine vision (recognition of the child, head to foot) and the size recognition work in combination.

### App Design and Technology

The size recognition of a patient and the dose calculation basically consist of three steps. In the first step, a person is detected in the camera’s field of view using an object recognition algorithm and is classified as a human being. In addition, the area in the image in which the child is located must be delimited as precisely as possible from the surroundings, and the measurement points (upper and lower limits) must be defined. In the second step, the distance between these two points and the camera is measured. This defines two points in 3D space, and the size can be calculated. In the final step, based on stored data, the respective dosages must be loaded and displayed.

#### Object Recognition

As soon as the app is started, it is ready to detect objects. The object detection is performed using the TensorFlow Object Detection API [26] and the TensorFlow Detection Model Zoo [27]. Based on different parameters, such as GitHub activity, Google searches, books, or job descriptions, it can be said that TensorFlow is the leading deep learning framework [28].

Recognizing people is one of the standard tasks of machine vision, so it should not be necessary to train the entire functionality from the beginning. To simplify the clarity of the app and to save resources, only the functionality for recognizing persons is activated. If a person is recognized, a bounding box is placed around this person and the confidence is displayed. With a confidence of 98% or more, the coordinates of the bounding box are stored in variables. The respective y-coordinates of two diagonally opposite points of the four corner points of the box indicate the size of the person.

#### Size Measurement

The size is measured using the Google Tango framework. Switching between the activities is done using the Intent class of Android. Tango is the predecessor of ARCore [29] and was developed for mobile phones with depth cameras. By default, Tango uses touch to set the measurement points manually. To automate this, the points from before are adopted. To prevent diagonal measurements, the x values of the two points are averaged and used as x-coordinates for both measuring points. It is important that both the object detection and the size measurement work with the same resolution (in this case, 1920×1080 pixels).

#### Dose Calculation

Depending on the size, the corresponding weight is loaded from a store and the appropriate dose is calculated and displayed using the formulas specified in the guidelines of the ERC [16]. The size to weight ratio is, as already mentioned, created using data from the KiGGS study of the RKI [25] and the formulas given in the guidelines of the ERC [26].

### Study Design and Measurements

**Figure 1:**
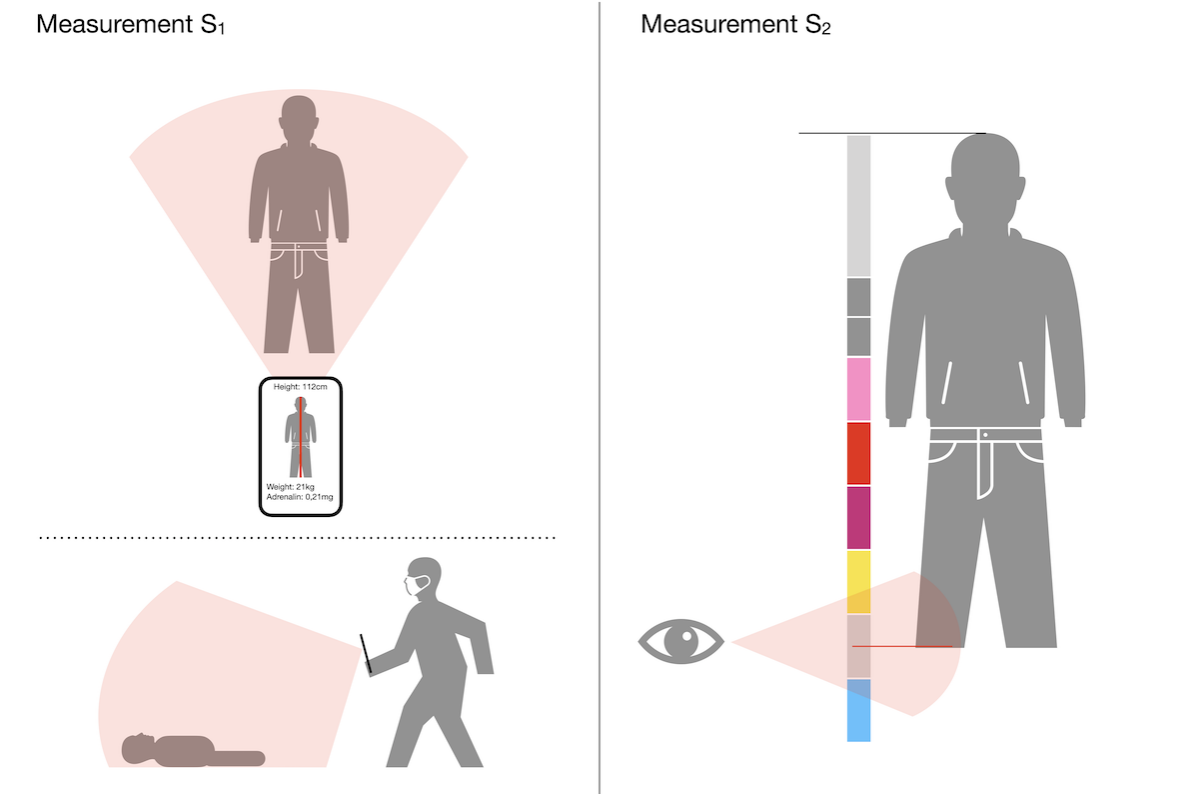
Schematic representation of the two measurements. S_1_, measurement with the app; S_2_, measurement with the emergency ruler.

The study design was a within-group setting in which each of the children was anonymously measured first with the app, installed on a ZenFone AR, and then with an emergency ruler (Pediatape [30]). The measurements (S_1_, S_2_) were performed in a room of a kindergarten during daylight. The children were lying on a wooden floor. For the measurement with the app (S_1_), the person taking the measurement stood in front of the child; the angle or the height of the camera was not specified. The process was similar to taking a photo with the smartphone. The only important factor was that the person being measured was captured as a whole by the camera (see Figure 1). When measuring with the emergency ruler (S_2_), the beginning of the measurement was placed at the head and the size was then read at the foot (see Figure 1). For both measurements, it was important that the person did not curve their legs. The parameters of age, height, weight, and gender of the children were not known from the beginning and were therefore randomly selected. In conclusion, there was one independent variable (measuring device) that took two values (app, emergency ruler).

### Recruitment

The study included 17 children from two kindergartens. Originally, over 50 parent letters were issued, but at the time of the study, only 17 permissions were obtained. There was no formal sample size calculation; the analysis plan was decided post hoc.

### Legal Aspects

Because the measurements performed could not cause any definable harm, no ethics committee was consulted prior to the execution. A legal review of the app with regard to the Medical Devices Act is pending but is considered to be too early at this stage.

### Statistical Analysis

#### Shapiro-Wilk Test

Because fewer than 30 subjects were available and we could not rely on the central limit theorem, which states that a sample is normally distributed when more than 30 samples are present, a Shapiro-Wilk test was performed prior to the statistical test selection to check for a normal distribution of the collected data.

#### Descriptive Statistical Analysis

To better understand the data before performing a statistical test, some descriptive statistics were calculated and plotted. These include the average of the respective measurements (S_1_, S_2_), the median, and the standard deviation. In addition, it was checked whether the expected high correlation between the related measurements and the expected correlation of the measurements with the corresponding weight of the children was actually present. Furthermore, the measured heights are presented with the corresponding weights.

#### One-Sample t Test

To test for a significant difference, a one-sample *t* test comparing the mean deviation of the difference (S_1_ – S_2_) to the reference value zero was executed. Two measurements are identical if the difference of the individual measurements is zero. The following hypothesis was tested at the significance level α=.05:

H_0_: There is no difference between the augmented reality application with automated size recognition using machine vision on the ZenFone AR and the Pediatape emergency ruler in the quality of the measurements.

If there were substantial differences, the app would not be suitable to act as an automated AR emergency ruler.

#### Bland-Altman Limits of Agreement

For a better overview, the results were additionally presented graphically. For this purpose, the Bland-Altman plot was used [31,32]. Thereby, the differences (delta) S_1_ – S_2_ of the individual measurements (S_1_, S_2_) were plotted against their mean ((S_1_ + S_2_)/2). This resulted in the following formula:



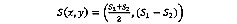



The upper and lower limits of agreement (LOA) are defined as 
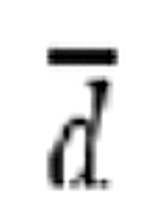
 ± 1.96*s* at a significance level of α=.05, where 
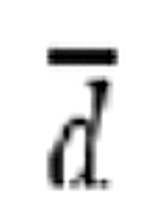
 represents the mean difference and *s* the standard deviation of pairwise differences. If 95% of the measurements lie within the LOA, both methods can be considered interchangeable, that is, both methods are equally appropriate [32].

#### Regression Analysis

To exclude a proportional bias, a linear regression analysis was performed at the end with the dependent variable (the measurement difference) and the independent variable (the mean).

## Results

Table 1 lists the measurements performed and the associated weight, sorted ascending by the height, measured with the app. If the height was somewhere between x.3 and x.7, x.5 was used; otherwise, the nearest full centimeter was used. This level of ambiguity is not a problem in the tested use case.

**Table 1:** Participants’ characteristics.

Participant no.	Height measured with the app (cm)	Height measured with the emergency ruler (cm)	Weight (kg)
1	98	98	15.1
2	98.5	97.5	15.3
3	99	101	15.3
4	99.5	98.5	15.4
5	100	100	18.9
6	101	105	20.2
7	102	100	15.1
8	104.5	105.5	19.4
9	107	107	19.4
10	111	113	21
11	111.5	112.5	22.6
12	112.5	113.5	21.5
13	113.5	112.5	20.5
14	113.5	116.5	23.1
15	119	118	20.8
16	119	117	23.1
17	121	119	19.4

### Shapiro-Wilk Test

Due to the small sample size, before applying a *t* test, it must be examined whether the sample is normally distributed. The difference (S_1_ – S_2_) and the mean ((S_1_ + S_2_)/2) were tested. The test was carried out using SPSS (IBM Corporation).

As Table 2 shows, none of the *P* values are smaller than the significance level α=.05 (*P*_diff_=.38; *P*_mean_=.06). It can therefore be assumed that the sample is normally distributed in terms of both difference and mean.

**Table 2:** Shapiro-Wilk test results.

	Statistic	*W*	*P* value
Difference	.95	17	.38
Mean	.90	17	.06

### Descriptive Statistical Analysis

Measured with the emergency ruler, the subjects are on average ø_er_≈108 cm tall with a median of 
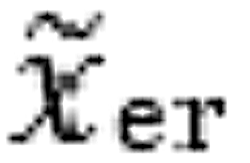
=107 cm and a standard deviation of *s*_er_=7.88 cm. The app comes to an average of ø_app_≈107.5 cm, also with a median of 
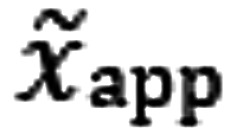
=107 cm and a standard deviation of *s*_app_=7.82 cm (see Table 3).

**Figure 2:**
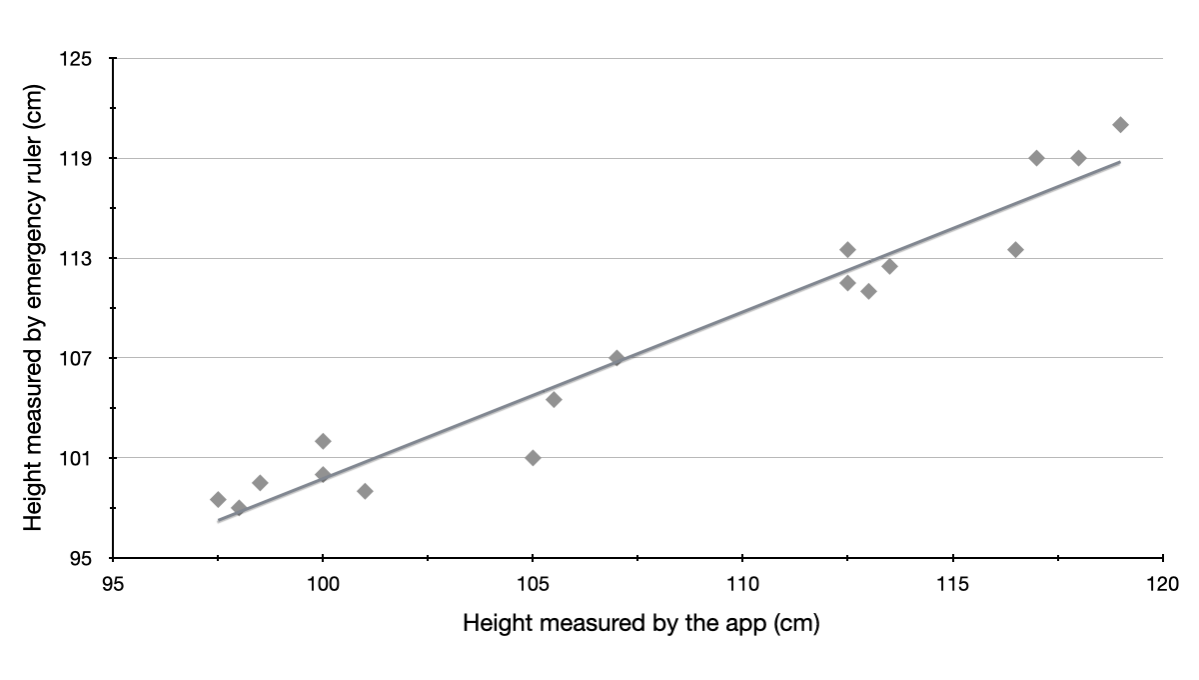
Correlation between the measurements taken with the emergency ruler and the app.

Table 4 and Figure 2 show a strong correlation between both measurements, as expected.

**Figure 3:**
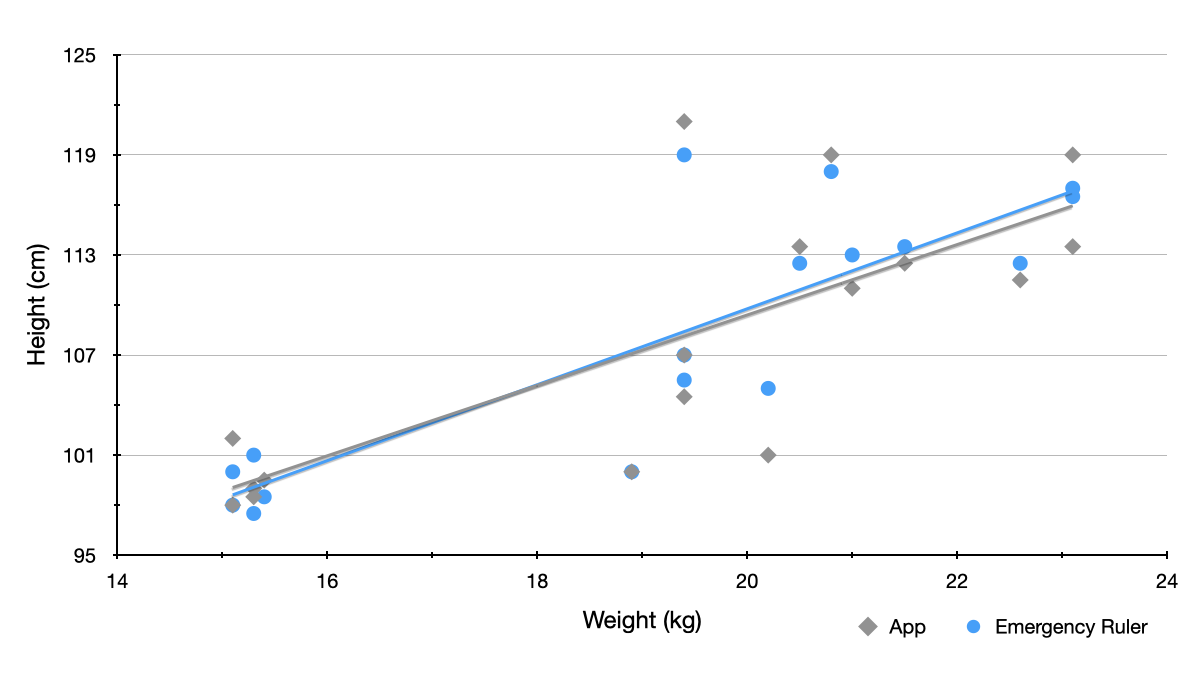
Correlation between both measured heights and the weight.

There is also a strong correlation between the measured heights (S_1_, S_2_) and the associated weights (Table 5, Table 6, and Figure 3).

**Table 3:** Descriptive statistics.

	Ø (cm)	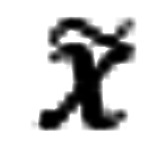 (cm)	*s* (cm)
Emergency ruler	108	107	7.88
Augmented reality app	107.5	107	7.82

**Table 4:** Correlation analysis (Pearson *r* and significance) between the measured heights using the app (S_1_) and ruler (S_2_) (N=17).

Variable		Height measured by emergency ruler	Height measured by the app	
**Height measured by emergency ruler**				
	*r*	1		.98
	*P* value	—^a^		<.001^b^
**Height measured by the app**				
	*r*	.98		1
	*P* value	<.001^b^		—

^a^Not applicable.

^b^The correlation is significant at a significance level of .05 (two-tailed).

**Table 5:** Correlation analysis (Pearson *r* and significance) between the measured height using the app and the weight.

Variable			Height measured by emergency ruler		Weight
**Height measured by emergency ruler**					
	*r*	1		.85	
	*P* value	—^a^		<.001^b^	
**Weight**					
	*r*	.85		1	
	*P* value	<.001		—	

^a^Not applicable.

^b^The correlation is significant at a significance level of .05 (two-tailed).

**Table 6:** Correlation analysis (Pearson *r* and significance) between the measured height using emergency ruler and the weight.

Variable			Height measured by emergency ruler		Weight
**Height measured by emergency ruler**					
	*r*	1		.77	
	*P* value	—^a^		<.001^b^	
**Weight**					
	*r*	.77		1	
	*P* value	<.001		—	

^a^Not applicable.

^b^The correlation is significant at a significance level of .05 (two-tailed).

### One-Sample *t* Test

To determine a significant difference between the two measurements, a one-sample *t* test of the measurement difference variable to the reference value zero was performed.

There was no significant difference in the measurement quality of the two measurements, according to the one-sample *t* test (t_16_=.82, *P=*.42; mean difference .35, 95% CI –0.56 to 1.26). The delta value of the measurements was not significantly different from the reference value zero (
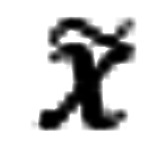
=.35, *s*=1.77). H_0_ can thus be retained.

### Bland-Altman LOA

**Figure 4:**
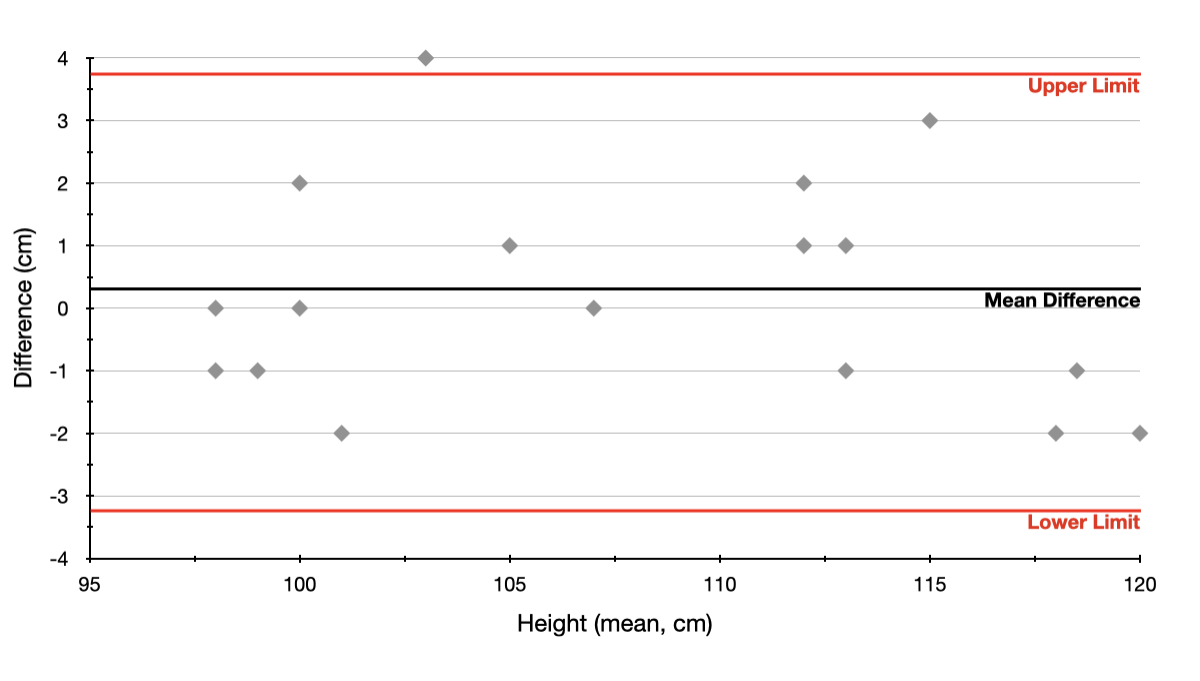
Bland-Altman plot.

In addition to the *t* test, the results are presented with Bland-Altman LOA values to provide a better overview. As can be seen in Figure 4, at least 95% of the measurements lie between the upper and lower limits. Therefore, it can be said that there is no significant difference in the quality of the two measurements to the significance level α=.05 and that both measurement methods are therefore interchangeable.

In most cases (9/17, 53%), there was a deviation of 0 to 1 cm. In 14/17 cases (82%), the deviation was 2 cm or less. Deviations with more than 3 cm were rare (1/17, 6%).

### Regression Analysis

**Figure 5:**
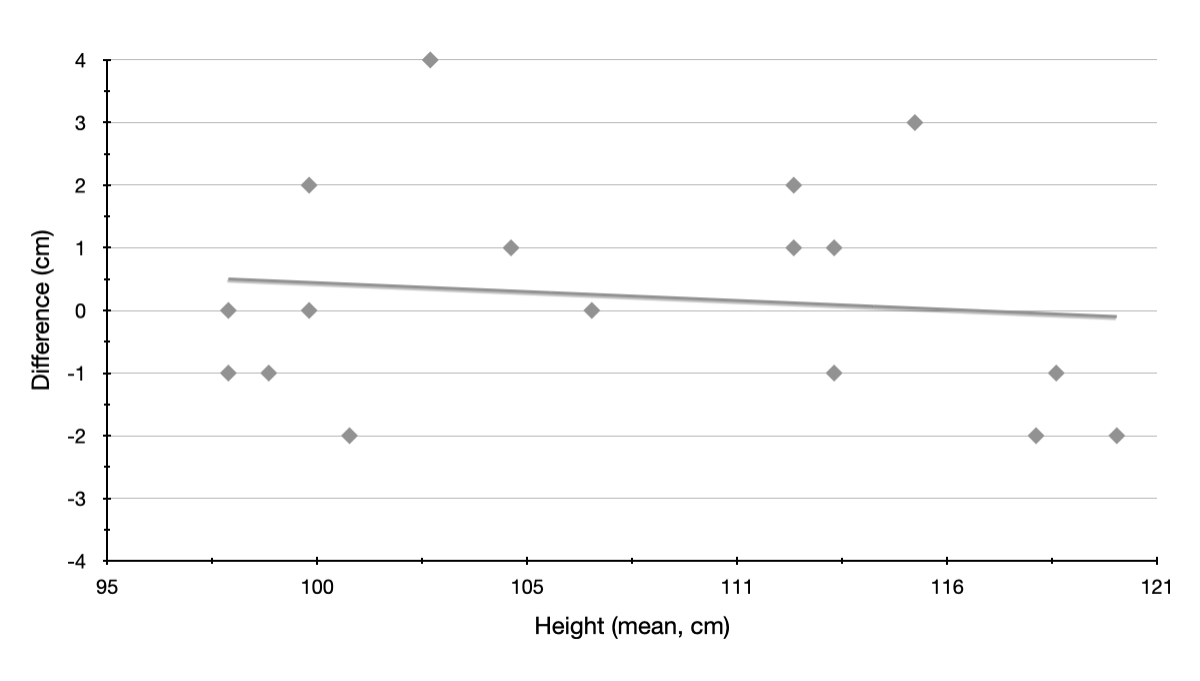
Regression line to exclude proportional bias.

Table 7 shows no statistically significant result. The beta value for the mean is close to zero; the *t* value is not significant. A proportional bias can be excluded. Thus, the app works the same regardless of the size of the children in the sample. For a better understanding, the regression line is plotted in Figure 5.

**Table 7:** Regression coefficients.

	Unstandardized		Standardized		
Model	Beta	SE	Beta	*t* (df)	*P* value
Constant	3.18	6.33	N/A^a^	0.50 (15)	.63
Mean	–0.03	0.06	–0.12	–0.47 (15)	.65

^a^N/A: not applicable.

## Discussion

According to this study, there is no significant difference in the quality of the measurements between a state-of-the-art emergency ruler and a smartphone with the newly developed app. The development of an assistance service including an AR emergency ruler is therefore technically possible. The *t* test indicates no significant difference (t_16_=.82; *P=*.42; mean difference .35, 95% CI –0.56 to 1.26). As shown in Figure 4, in more than half of the cases (9/17, 53%) the differences are 1 cm or less. The largest deviation is 4 cm. The sample size is admittedly rather small, with 17 normally distributed samples, but the results suggest that the distribution will not change with larger sample sizes. For quality assurance purposes, only the weight was collected in addition to the two measurements. Age and gender were not included due to the principle of data minimization and the promised protection of anonymity. This means that it should not be necessary to calculate BMI retrospectively. Although this would be theoretically feasible, for the interpretation both age and gender are necessary in children [33]. Therefore, no classification of weight can be made. It should be further noted that the measurements were carried out in an indoor environment in daylight. Especially strong light or a bright background might affect the infrared camera. It should also be noted that Google discontinued its ambitious “Tango” project in 2018 [34], which was used for this app. Due to the additionally required expensive hardware (depth camera with infrared sensor), the Tango project could not become generally accepted. For AR game apps, it is not important how faultlessly objects are placed in space. Therefore, Google has decided on a type of technology that runs on almost all smartphones (ARCore). However, the Tango API is still available in Google Archive [35]. The latest trend in AR, however, is again moving toward depth sensing, as shown by Microsoft with the HoloLens [36], by Samsung with the Galaxy S20+ [37], or by Apple’s new iPhone 12 Pro with light detection and ranging technology [38]. The extent to which these implementations are suitable for continuing this research needs to be evaluated. The long-term goal of this research is to create a ubiquitous pediatric emergency assistance service. An important step has been achieved with the automatic size recognition. At the moment, a study at a simulation center is planned to explore the usability aspects of the app under the most realistic conditions possible.

## Abbreviations

AR: augmented reality
ERC: European Resuscitation Council
HMD: head-mounted display
KiGGS: Studie zur Gesundheit von Kindern und Jugendlichen in Deutschland (German Health Interview and Examination Survey for Children and Adolescents)
LOA: limit of agreement
RKI: Robert Koch Institute
